# The Independent Practitioner

**Published:** 1888-02

**Authors:** 


### THE INDEPENDENT PRACTITIONER.

Our many friends will be glad to know that this edition of the Independent Practitioner is very much the largest that it has ever issued. From the beginning there has been a steady and healthy growth in its circulation, and at the commencement of each half year since its present publishers have had its management, it has been found necessary to enlarge the edition. Until the present time, it has never made any special effort to increase its subscription list. It has offered no premiums or other special inducements to obtain subscribers, preferring to have its increase depend solely on its merits. The present offer of Prof. Stowell's great portfolio was prompted, not so much for the purpose of helping this Journal as by the desire to fulfill all that is incumbent upon true professional journalism, to spread information by every attainable means, and to familiarize every dentist with the histological structure of the organs upon which he works.

If the publishers of this Journal have an honest and earnest ambition, it is to be of some real service in their profession, and to this end they have devoted money and labor freely, without the hope or expectation of pecuniary reward. They are proud to say that their efforts have met with a recognition both ready and hearty. We know that the great majority of dentists, in this and other countries, will rejoice at the success and continued prosperity of this Journal, and we earnestly hope that all of our worthy contemporaries may enjoy equal success. The Independent Practitioner has never permitted itself to indulge any feeling of jealousy towards its compeers, but hails them all as co-workers with it in the great cause of dental progress.

